# Evaluating the contribution of shape attributes to recognition using the minimal transient discrete cue protocol

**DOI:** 10.1186/1744-9081-8-53

**Published:** 2012-11-12

**Authors:** Ernest Greene, R Todd Ogden

**Affiliations:** 1 Department of Psychology, Laboratory for Neurometric Research, University of Southern California, Los Angeles, CA, 90089-1061, USA; 2 Department of Biostatistics, Columbia University, New York, NY, 10032, USA

**Keywords:** Shape recognition, Contour attributes, Shape encoding

## Abstract

Subjects were tested for their ability to identify objects that were represented by an array of dots that marked the major contours, usually only the outer boundary. Each dot was briefly flashed to make its position known, and a major variable was the time interval that was required to flash all the dots for a given shape. Recognition declined as the total time for display of the dot inventory was increased. Each shape was shown to a given subject only once and it was either recognized -- named – or not. Although the recorded response was binary, a large number of subjects was tested, which made it possible to derive regression functions and thus specify an intercept and slope for each shape. Shapes differed substantially in their slopes, which is likely due to the amount of redundant information provided by neighboring dots. Indices of shape attributes were also derived, specifically Attneave’s indices of complexity, mean curvature, inflection count, and symmetry. Three of the four shape attributes were significantly related to intercept and slope levels, but none made a substantial contribution. This suggests that these attributes are not essential properties that define shapes and allow for recognition.

## Background

**Figure 1:**
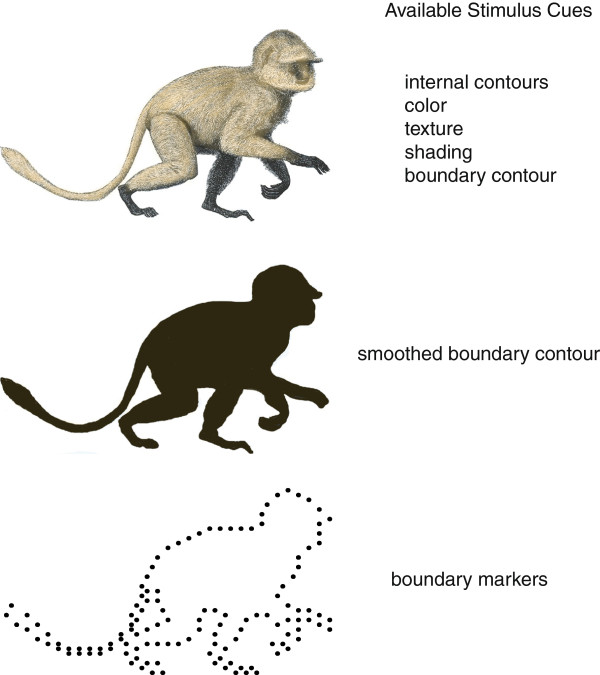
**The upper panel shows a number of cues that may contribute to recognition of a given object.** Although 3D cues are not available in this stimulus, the color, texture, shading, internal contours, and boundary contours all allow the monkey to be identified. The silhouette in the middle panel provides only the boundary contours, but this is still adequate for recognition. The bottom panel has replaced the boundary contours with an array of dots. This stimulus is sufficient for recognition with substantial reliability even when the dots are displayed for only a brief moment. It is possible that identification of the shape does not depend on registering collinearity of the dots that are serving as contour markers.

Numerous stimulus cues can be useful for identifying objects. Distinctive coloration, texture and depth cues contribute to our ability to distinguish one object from another [1-5]. The lines and edges of the object, *i.e.,* the contours, are considered to be especially critical [6-8]. In many cases it is possible to discard all cues other than the outer boundary of an object, as shown by the silhouette in Figure 1, and the object can still be identified [9].

One can further probe the object recognition system by reducing the cues even more. In the lower panel of Figure 1 the boundary contour has been replaced by a string of dots, yet one can still recognize the stimulus as having the shape of a monkey. Here the dots have fairly close spacing that follow the path of the boundary, and one might presume that recognition of the shape depends on the nervous system being able to register alignment among a set of adjacent dots much as it would respond to a continuous contour.

However, previous work from this laboratory has suggested otherwise. Greene [10] found that shapes could be identified with display of very few evenly spaced dots that marked the outer boundary. The location of dots in the pattern were not chosen to mark specific features of the shapes, such as major inflection points, and sparse dot patterns allowed for recognition of asymmetrical as well as symmetrical shapes. Based on this and related evidence, Greene [10,11] has proposed: a) that the dots can serve as independent markers of locations within contour segments, and b) distances among these markers or from the markers to a centroid provide “metric” information that is summarized and provides a basis for shape (and thus object) recognition.

One can challenge the perceptual system even more by presenting each dot for only a very brief instant or with successive display of the inventory of dots that mark the boundary. This has previously been designated as the minimal transient discrete cue (MTDC) protocol [11-16]. The brief flash of a given dot activates the retina, providing a small amount of information that persists for a limited amount of time. Recognition of a given shape depends on being able to combine across the aggregate of traces provided by the successive dots, and the odds of being successful declines as a function of the total time interval for display of the dot inventory. Being able to better specify the rate of decline for various shapes should provide more insight into how the individual cues combine for purposes of recognition.

For the present study, we tested a large inventory of shapes using the MTDC protocol, varying the total time required to display all dots that mark the boundary of each shape. A large enough number of subjects was tested to allow the rate of decline to be established for each shape in the inventory. Across shapes, we observed major differences in this rate of decline. It seems unlikely that these differences in slope were due to differential salience of individual dots, or shape-specific factors that would modify information persistence. We provide evidence that the differences in the rate at which recognition declines is determined by the amount of information redundancy among the dots of a given shape.

We also examined whether shape characteristics, specifically the ratio of the perimeter length to the enclosed area, amount of curvature, number of inflection points, and symmetry were predictive of the recognition-potential of a given shape. Although some significant relationships were found, none accounted for much of the total variance. This weighs against theories that these attributes define a given shape and determine how readily it can be identified.

## Methods

### Stimulus display board

Display sets were presented to the observer by brief emissions from a 64 × 64 array of LEDs, designated as the display board. This board differed in several respects from one previously used by this laboratory, most notably in being able to display any number of dots simultaneously. The array was comprised of AlGaInP LEDs, specifically RL5-R8030 (Super Bright LEDs, Inc.), which have a wavelength of 630 nm (red). The diameter of each LED was 5 mm, center-to-center spacing of the array was 9.4 mm, and the array measured 60 cm between the centers of outer elements in both the horizontal and vertical directions.

The plane of the array was tangent to the line of sight of the observer, and positioned at a distance of 3.5 m. From this distance LED diameters and center-to-center distances were 4.92 and 9.23 minutes of visual angle, respectively, and the full array was 9.80 × 9.80 degrees of visual angle. With the diameter being less than 5 minutes of visual angle, it is appropriate to consider each LED to be a point source, and thus to specify brightness as luminous intensity, as detailed below.

The stimulus display was delivered from a Propox MMnet101 microcontroller that ran at a clock speed of 16 Mhz. Firmware instructions were processed at an average speed of 12 MIPs. This system allowed for control of pulse duration with a maximum error of 1 μs. Instructions to the microcontroller were provided by a Mac G4 Cube, which specified experimental protocols using Tcl/tk custom applications written for OS-X.

### LED emission and ambient lighting

Brightness of LED emissions was set by controlling the voltage level that was applied, with a 220 ohm resister being placed in series with each LED. The manufacturer provided a number of measures of luminous intensity as a function of voltage. These values fit the equation Cd = 0.17492*V^2^ + 0.17637*V - 1.0634, which provided an extended scale of control values. Luminous intensity of LED emissions was set at 1 Cd using this formula. Rise time (10%-to-90%) and fall time (90%-to-10%) for emission was 200 nanoseconds.

Standard fluorescent fixtures were fitted with occluding panels so that intensity of room lighting could be controlled without changing color balance. The occluders were positioned to provide 10 lux of ambient illumination as measured using a calibrated Tektronix J 1811 photometer. At this light level the dark gray matrix within which the LEDs were mounted appeared black.

### Calibration of shapes

Shape stimuli were derived from the Macmillan Visual Dictionary [17], from Hemera Photo-Object files, and from images that were sampled from the Internet. As was true for earlier work [10-16], a great many of the shapes were animals, furniture, vehicles, and tools. In addition to these categories, the present inventory of shapes included distinctive human activities, cartoon characters, well-known iconic symbols, and truncated portions of objects, *e.g.,* heads, feet.

Discrete locations on the major contours, in particular on the outer boundary, were marked with the aid of a custom program. To mark these positions, each shape was adjusted for size by overlaying it with a 64 × 64 grid, which was expanded or contracted until it touched the outer boundary of the shape in either the horizontal or vertical direction. Then grid cells that included the major boundaries of the shape were “marked,” meaning that the address positions were recorded in a table. For convenience, these marked address positions may also be referred to as “dots.”

**Figure 2:**
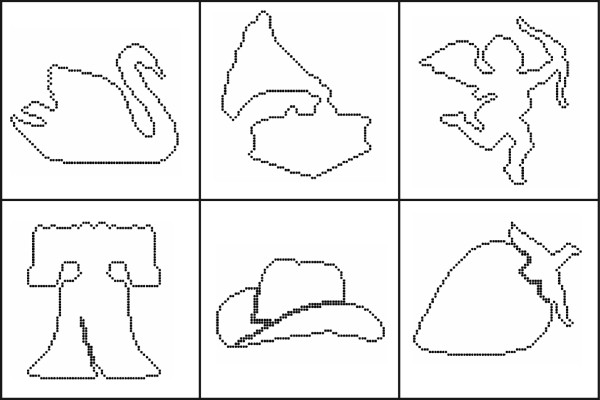
**The upper panels provide a sampling of shape patterns in which dots marked only the outer boundary of the shape.** Fourteen percent of the inventory had shapes with internal contour lines, as illustrated in the lower panels.

The panels of Figure 2 show typical shapes in several of the object categories. A great majority of the shape-patterns provided only the outer boundary, such as the swan, gramophone, and cupid. However, internal contours were included for 53 of the shapes, subject to the requirement that all dots be traceable along a path where each dot was encountered only once. The Liberty Bell, cowboy hat, and strawberry provide examples of shape patterns that included internal contours.

The major contours of 450 shapes were prepared in this manner, some of them being very similar alternative versions, as discussed below. The first goal was to determine which of these many shapes could be reliably named when briefly displayed. This was established using 10 subjects, each tested individually, who were seated opposite to the display board as described above. Each shape was shown one at a time by simultaneously flashing all of the dots in the address table for 50 μs. Subjects were asked to name each shape immediately after it had been displayed and a response was typically provided within a second or two. An acceptable set of alternative names had been decided upon in advance of testing. However, in some instances the subjects offered names that were deemed to be reasonable, and these were added to the list of acceptable names.

Of the initial 450 candidate shapes, a great majority was identified by all ten of the subjects. Testing could have been restricted to just this subset, but it was decided to include all shapes that had at least a 50% level of recognition (hit rate), to allow comparisons that covered a larger range of recognition difficulty.

As indicated above, two or three alternative versions of some objects were scanned and discretized, on the theory that the one showing the most consistent recognition function would be used in subsequent experiments. Having reached the point where this decision should be made, the shapes themselves, along with their respective raw-data plots, were examined. For eight pairs, one member was rejected as being too similar to the other member and having a comparable level of potential for recognition. One member of a triplet was also rejected on this basis. However, in all other cases, there were sufficient differences in the contours being displayed that it seemed prudent to retain each version of the shape.

After removing the 9 redundant shapes from those that had a 50% minimum hit rate, 376 shapes remained in the inventory. The number of shapes having hit rates at 100%, 90%, 80%, 70%, 60% and 50% were 175, 70, 44, 30, 31, and 26, respectively. Mean hit rate for this inventory was 87%. These shapes are listed in Additional file 1: Table SA along with the hit-rate and the number of dots in the address table of each shape.

### Display timing conditions

For display of a given shape, all dots listed in the address table were displayed, each dot emitting light for 50 μs, which is designated the T1 interval. Hereafter this brief emission from a dot is described as a “flash”.

One treatment condition displayed all dots in the address table at the same moment. Although this condition provided for a total time of display of 50 μs, it is nominally designated as TT = 0.

Three other treatments displayed one dot at a time, with the total time (TT) for display being 100, 300, or 700 ms. This was accomplished by adjusting stimulus onset asynchrony (SOA) -- the time from the beginning of one flash to the next. For each shape, the SOA was calculated by dividing total time (100, 300, or 700 ms) by the number of dots to be displayed. Thus when all the dots were presented in succession using the SOA that was appropriate for a given shape, all of the dots were delivered within the total time that the condition required.

**Figure 3:**
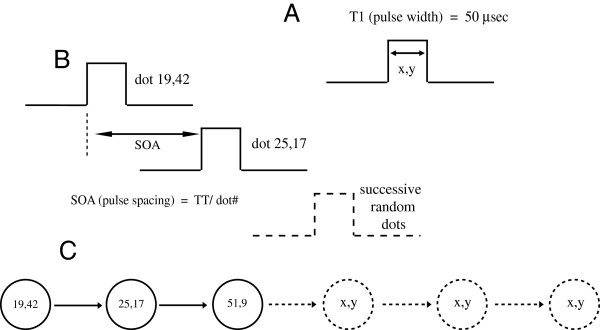
**A. Each dot was addressed using a Cartesian (x,y) coordinate system.** For each dot serving as a boundary marker, its position was made known by flashing it just once for 50 microseconds. **B.** At TT = 0, dots that marked the boundary were flashed simultaneously . However, at TT = 100, 300, and 700 milliseconds dot positions were selected at random and flashed one at a time. For a given shape, the interval between successive dots, *i.e.*, the SOA, was the total time provided for display of all dots divided by the number of dots for that shape. **C.** Successive display of dots is indicated, with the dashed portion at the right of the sequence meaning to represent random choices of dots until all have been flashed. The final dot at the right of the sequence meant to represent random choices of dots until all were flashed.

Restating, there were four conditions that varied the total time across which all dots within the address table of each shape were displayed, these being: TT = 0, 100, 300, and 700 ms. The timing conditions are illustrated in Figure 3.

### Subjects and testing protocols

Eighty subjects were recruited from the USC Department of Psychology Subject Pool. Each subject judged the stimulus displays using both eyes, allowing correction with contact lenses or glasses as needed. Each was tested individually, being shown each of the 376 shapes only once and with each shape having been assigned to one of the TT conditions. A counterbalance was added that assured that each of the four treatments would be seen an equal number of times; across each successive four subjects that were tested, a given shape was randomly assigned to one of the four treatment conditions, without replacement.

For each of the timing conditions except TT = 0, dots were shown in a random order for each shape and this order differed for each subject. The assistant who was testing the subject had no information about what treatment was used for a given shape nor did this individual know the treatment conditions being tested.

Subjects responded by naming the shape that was shown, generally offering the name within one or two seconds. The experimenter then entered a keystroke to record whether or not the name was correct, scoring it as correct only if the name had been deemed acceptable during development of the shape inventory, as described above. Subjects were not told whether or not their responses were accurate. A test session generally lasted about 45–55 minutes, including a five-minute break after half the shapes had been displayed.

## Results

### Regression for individual shapes

As described in Methods, each subject saw a given shape only once, and provided a response that was classified as recognition (1) or no recognition (0). There were four TT conditions and 80 subjects, so for a given condition 20 subjects judged each shape. This allows the binary responses to be expressed as a hit rate for each of the TT conditions, *i.e.,* the total number of times the shape was recognized across the 20 times it was judged.

For binary response data the customary approach to testing for treatment effects involves fitting a logistic regression model. We fit a mixed effects logistic regression model to all the data with TT as a fixed effect and shape and subject as random effects. Application to the present data indicated that TT has a very highly significant effect on hit rate (p < 10^-15^), as shown in Table 1.

**Table 1:** Estimated parameters, standard errors, and significance tests for the fixed effects in the generalized linear mixed model fit with subject and shape as random effects

	**Estimate**	**Std. error**	**z value**	**p-value**
(Intercept)	2.2516	0.1049	21.47	<2 x 10^–16^
TT	-4.8662	0.1577	-30.86	<2 x 10^–16^

Although it is good to provide statistical confirmation of effects, there was never a question of whether the successive display would produce a decline in hit rate, for it had been reported by this lab in a number of prior studies (11–16). The reason for running so many subjects with the present treatment conditions was to provide enough data to calculate a regression function for each individual shape, thus providing indices that could be used as covariables or as predictors of effect with respect to shape attributes.

Prior research from this laboratory (11,14,16) has shown that successive display of the shape dots produces remarkably linear declines in recognition as a function of time differentials and ambient light levels. So for the purposes of deriving shape-specific indices of TT effect we calculated a linear model for hit rate as a function of TT and fit the data for each shape separately. A binomial response variable may be obtained for each shape and for each TT value by aggregating over subjects. Standard regression analysis with binomial responses will yield unbiased estimates of slope and intercept for each shape. But since binomial random variables do not have the same variance as required by usual regression analysis, estimation of slope and intercept may be improved by weighting each response by its estimated variance. We performed regression analysis for each shape using both weighted and unweighted procedures. The conclusions given in this section are based on the unweighted estimates, but we performed the same analyses using the weighted estimates and drew the same conclusions.

**Figure 4:**
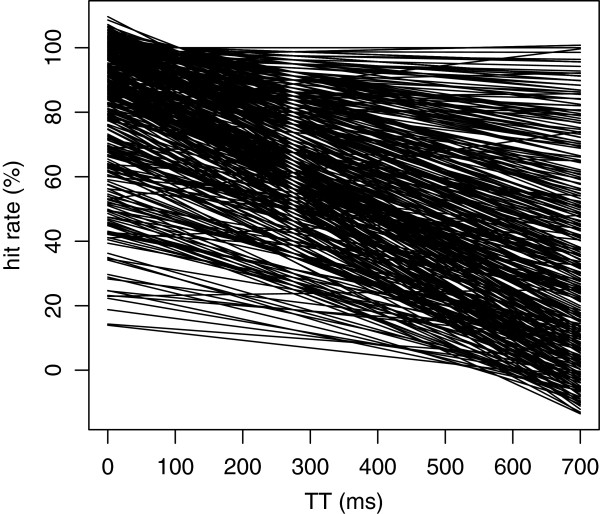
**A regression line was fit to the mean hit rate for each of the 376 shapes, and these lines have been plotted.** One can see a sizeable range of intercept values at TT = 0, and a large range of slopes.

Figure 4 shows the regression models that were calculated for each of the 376 shapes, and the intercept and slope for each of the shapes are listed in Additional file 1: Table SA. Very few of the fitted regression models extended above 100% or below 0%. All but a few manifested a decline in recognition as a function of TT.

**Figure 5:**
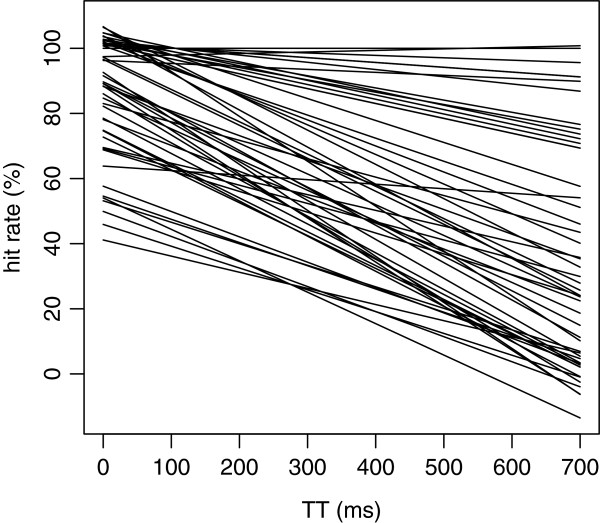
The regression lines from a random sample of 50 shapes have been plotted, the lower density making it easier to see the range of intercepts and slopes.

On the possibility that the density of the overlapping plots in Figure 4 fails to communicate the range differential of the slopes, Figure 5 shows regression lines from a random sampling of 50 shapes from the inventory.

**Figure 6:**
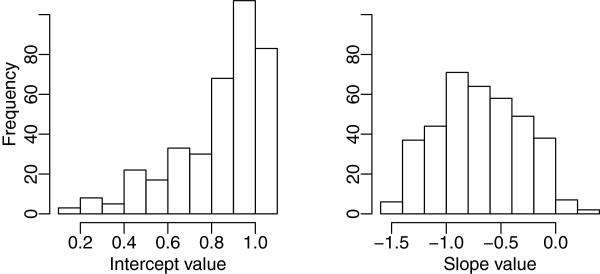
**The left panel shows the relative abundance of intercept values, *i.e.*, predicted hit rate at TT = 0.** A great majority of the shapes had intercepts at or above 80%. The right panel shows the frequency of regression slopes.

In Figures 4 and 5 one can see that the great majority of shapes had regression intercepts, *i.e.,* predicted recognition at TT = 0, of 80% or higher. This can also be seen in the left panel of Figure 6. In the right panel one can see the relative frequency of slopes, which comes much closer to being normally distributed.

**Figure 7:**
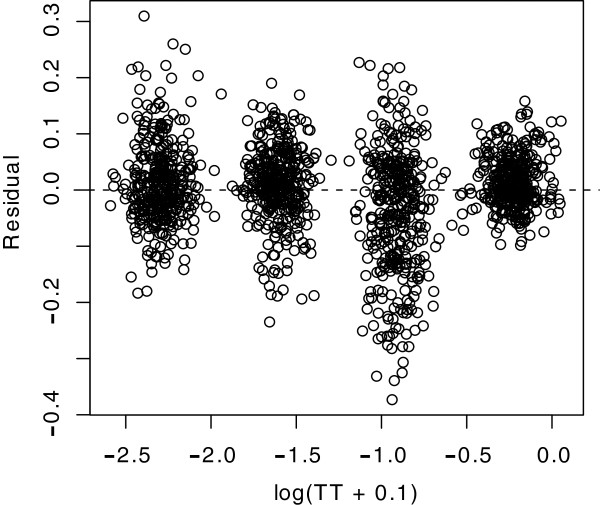
**Departures of hit rate from the values predicted by the regressions are plotted for each of the four treatment levels, each shape providing a plot-point for each of the treatments.** The relatively consistent balance of the clusters around zero suggests that there are no systematic nonlinear trends in the data.

Figure 7 shows a plot of the size and direction of departures from the linear model for each shape, *i.e.,* residuals with some random jittering on the x-axis so that points are more visible. In general the four clusters appear to be well balanced with respect to the regression models, *i.e.,* at 0, and there does not appear to be a systematic departure from linearity.

Previous research [16] found differentials in hit rate as a function of the number of dots being displayed. However, those results were obtained using a protocol in which the SOA for display of dots was the same irrespective of how many dots were used to mark the boundary. This meant that the total time of display for a given shape was greater for shapes having many dots than for those having few. This left open the possibility that the differentials were due to total display time. Here we adjusted the SOA according to the number of dots to be displayed, thus making the total time (at each TT level) the same for all shapes.

**Figure 8:**
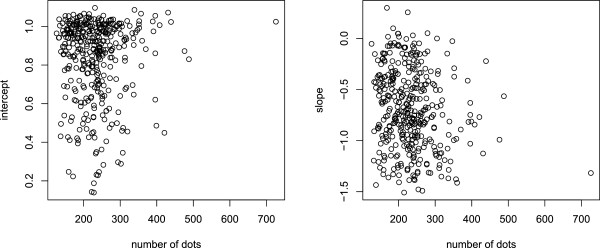
**In the left panel, a scatterplot was used to determine whether the number of dots being displayed for a given shape determined the size of the intercept, *i.e.*, the predicted hit rate at TT = 0.** The shape of the plot does not suggest a relationship. Likewise, the number of dots did not appear to influence the slopes of the regression functions (right panel).

The left panel of Figure 8 shows a scatterplot for the magnitude of the intercept for each shape as a function of the number of dots in each shape. The intercept reflects the difficulty level of the shape when it is displayed at TT = 0, and there was no indication that the number of dots determines how readily the shape can be identified. Note, however, that here each shape was displayed using the full inventory of dots. As discussed below, there is redundancy in the information provided by the dots and the number of dots becomes relevant if there is sparse sampling.

The right panel in Figure 8 plots the size of the slope as a function of dot number, and it does not appear that the number of dots contributes to the slope differentials.

Returning to the slope differentials plotted in Figure 4, a major unexpected finding of the present experiment was that shapes differed substantially in the rate of decline in recognition as a function of total time. Shapes that were comparable in baseline difficulty, as reflected in similar hit rates at TT = 0, differed substantially in the rate at which recognition declined at longer TT intervals. Some shapes, for example, dropped precipitously from 90-100% recognition at TT = 0, to hit-rates of 50% or less when the total time for display was 700 ms. Other shapes manifested a low slope, remaining in a narrow hit-rate range across the full time interval that was assessed, *i.e.,* 700 ms.

### Source of slope differentials

Explaining the slope differentials requires adopting a model of the process by which the dot information is combined to yield recognition, or at least offering a tentative hypothesis. Each dot provides a small amount of information about the location of the shape boundary and one must combine the information from many dots in order to identify a given shape. When the dots are presented briefly, as was done here, the information is thought to persist for a short time, and the ability to identify the shape depends on being able to integrate across information that has not yet decayed.

It seems unlikely that the duration of information persistence for a given dot would vary as a function of the shape to which the dot belongs, and the scatterplots in Figure 8 do not suggest that the number of dots being displayed are factors in how much information can be integrated over a given time period. What seems more plausible is that the net density of dots required for recognition differs across shapes, with a greater proportion being needed for some shapes than others. This was demonstrated in an earlier study from this lab, in which subjects were asked to identify shapes that were displayed with continuous illumination of the dots (not as brief flashes), but where only a sparse sampling of the full inventory of boundary dots was shown [10]. Recognition of some shapes was possible with display of only a small portion of the dots, whereas other shapes required a much larger percentage. We can describe the essential fraction as a “critical density”.

This leads to the hypothesis that shape identification that requires a low critical density will have a lower slope than one that requires a greater critical density. Dots that are in close proximity provide much the same information about the location of the boundary, and are essentially redundant. With dots being picked at random for rapid sequential display, the critical density needed for recognition will be available within a time interval that is only a portion of the total display time. If the shape requires a low critical density of dots, the number needed for recognition will be present early in the display sequence, and also through the middle of the sequence, and again at the end of the display period. The process by which the shape is summarized thus gets multiple (or continuing) bites at the apple, so to speak.

We were able to evaluate this hypothesis making use of some unreported data that asked for recognition when shapes were displayed using sparse sampling of the dots. In that study the shapes were continuously displayed until the subject responded. The criterion for choosing which of the original 450 shapes to display was different, but 340 of the shapes were the same as those in the present study. Ten subjects had been tested with 100% of the dots being displayed, and another 20 subjects had been tested with sparse dot densities. The sparse displays provided every 3^rd^, 5^th^, 8^th^, or 12^th^ dot from the address table, this being 33%, 20%, 12.5%, and 8.3% of the total dot inventory, respectively. Given that there were 20 subjects and four sparse treatment levels, five subjects contributed decisions at each treatment level.

**Figure 9:**
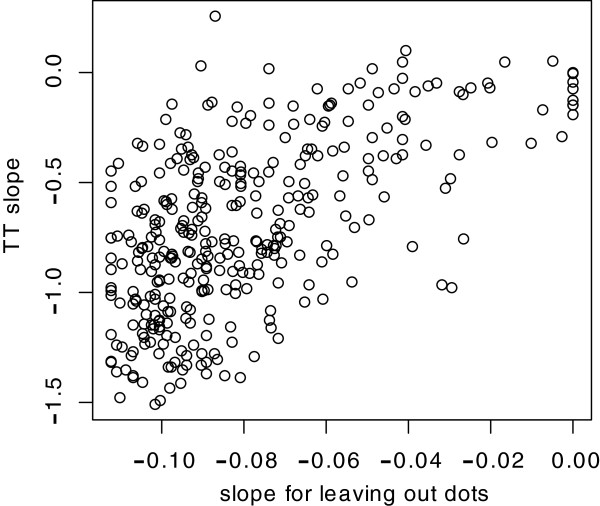
**A large portion of the shapes that were tested with variation of TT had also been tested with various degrees of sparseness, *i.e.*, display of only some of the full inventory of boundary dots.** Hit rates decline when fewer dots are displayed, and the slope of the regression on a given shape reflects the degree to which the dots are providing redundant information for purposes of recognition. Here the slopes of the sparseness regressions are plotted against the slopes of the TT regressions. There is a clear relationship, which supports the proposition that the variation of slope that can be seen in Figures 3 and 4 are due to differences in the degree to which neighboring dots provide redundant information.

Regression lines were fit to the hit rates that were observed as a function of the five levels of dot-density (sparseness). The slope of each regression line specifies the degree to which the dots are redundant and our hypothesis is that this determines, or at least contributes to, the degree to which recognition declines as a function of TT. Figure 9 plots the slopes that were observed for the 340 shapes as a function of dot-density, against the slopes that were observed across the range of TT intervals.

The correlation coefficient for these data is 0.60, and the percent of variance accounted for by a linear regression, *i.e.,* R^2^, is 0.36. A permutation test for the significance of this relationship gives p < 0.001. Repeating the analysis on slopes estimated using binomial-based weightings gives essentially the same conclusions (R = 0.60; p < 0.001).

These results are consistent with the concept that the rate of decline in hit rate for a given shape depends on the degree to which neighboring dots are redundant. If they have substantial redundancy, the shape can be identified even if dots are removed. In like manner, when the dots are successively displayed, each being briefly flashed, the hit rate depends on integration of information that is transient and quickly becomes unavailable. The slope of the hit-rate function therefore depends on how much information is needed for recognition, and it does not decline as quickly if the essential information can be provided by only a small percentage of the dot inventory. Thus TT slopes will be steep if a larger percentage of dots are required for recognition, and shallow when only a small percentage is needed.

### Additional consideration of redundancy

Neighboring dots and dot residuals can be considered as redundant if they provide essentially the same information about the shape to be identified. One of the simplest examples would be a circle that can be recognized with display of a very small complement of equally spaced dots. With continuous display of sparse, evenly spaced dots, Greene [10] found that almost a fifth of the shapes could be identified when the density of dots was below 10%. These included shapes that were asymmetrical, *e.g.,* boot, pipe, rooster, banana, hat, man's shoe, cow, spoon, hen, and woman's shoe. A moth, rooster, woman's shoe, and boot required only 21, 19, 13 and 11 dots, respectively. This was the average across subjects, and some of the 18 subjects of that experiment identified the shapes with fewer dots, even though the odds of doing so on the basis of chance guessing was only 0.89%.

It is unlikely that contour encoding systems of the brain, *i.e.,* receptive fields of orientation selective neurons [18,19], could register the shape boundaries with these extremely sparse dot patterns as the only cues. Sceniak et al. [20] used drifting sinusoidal gratings to stimulate simple and complex neurons in primary visual cortex of macaque monkeys. They found the length of the excitatory field for 30 of the 31 fields that were examined to be shorter than 2.5 degrees of visual angle. Greene [10] tested three subjects displaying every fifth dot and positioning the subjects so that the minimum visual angle between successive dots was 2.5 degrees. These subjects had hit rates of 33, 64 and 68%, where the odds of successful identification by guessing was only 0.89%. Therefore, recognition of shapes is possible when the dot-to-dot distances exceed the size of receptive fields of orientation-selective cells. One might speculate about a role for long-distance connections within the cortex, but these serve only to combine and tailor primary responses.

Further, successive display of short arrays of dots that should activate the receptive fields is no more effective at eliciting recognition than is display of the same number of dots that lie at randomly selected positions [11]. For the many instances where a small number of widely spaced dots were found to be sufficient for recognition of the shape, it is difficult to specify a path-rule by which the boundary would be reconstructed. For these several reasons, Greene [11] has argued that shapes (and thus objects) can be identified on the basis of the metric information provided by dots that are acting individually as boundary markers.

Three of the treatment conditions presented the entire dot inventory for each shape, randomly ordered, and briefly displayed one-at-a-time. It is likely that a large percentage of the dots were essentially redundant in marking the location of the shape boundary, and recognition was possible when only a fraction of the full inventory was shown. This would allow for recognition even at relatively long display times. Though many or most of the dot residuals would have decayed and were no longer serving as useful markers, some would remain effective as shape cues. In other words, the slope of the decline in recognition as a function of total time would be less steep if most of the dots were redundant. Our supplemental data analysis indicated that shapes having a low total-time slope also had a low slope with respect to the impact of removing dots from the display.

### Evaluating shape attributes

Many factors affect how readily an object can be identified, including familiarity and the number of alternatives to be distinguished. Recognition may depend on physical characteristics such as color and texture. However, the geometry of the major contours, and especially the outer boundary, is considered to be especially important for object recognition. These contours provide what we generally describe as the “shape” of the object, and here we direct our attention to whether various shape attributes are important for recognition.

Fred Attneave (1919–1991) was not the first to discuss curvature and angles as critical shape attributes, but he is generally acknowledged as being one of the first to discuss these attributes in terms of information content. He specified that the points at which the boundary of a shape change more quickly provide the major information that defines the shape [6]. To demonstrate this point, he showed subjects silhouettes of amorphous shapes, and then instructed them to mark 10 dots on blank pages to “reproduce” each shape. He found that dots were far more likely to be placed at the boundary locations that had the greatest change in curvature.

Subsequent experiments [7] supported this point and provided quantitative measures of shape complexity. Here he constructed shapes, starting with random points and then adding connecting lines and curves to provide an outer boundary. He specified a size invariant measure of “complexity” of the shapes, this being the ratio of the square of the perimeter divided by the area – P^2^/A – and found that this index was highly correlated with subjective judgments of shape complexity. An index of symmetry was not a strong predictor, which may be a function of the kind of shapes that were created by his protocol. The contour attribute that was most predictive of subjective assessment of shape complexity was the number of turns, *e.g.*, the apices of angles, the maxima of convex curves, or minima of concave curves.

Norman et al. [21] essentially replicated the Attneave [6] study, confirming that dots that are placed near maxima or minima provide an “optimal” copy of the figure. Feldman & Singh [22] submit that the negative extrema (which we are calling minima) provide more information than do the positive extrema (maxima), based on the argument that natural objects are generally more convex than concave. De Winter & Wagemans [23] examined the question of what locations on silhouettes of everyday objects are considered by subjects to be the most salient. They found that 85% of the points chosen were closer to the convex maxima or concave minima than at the transition from one to the other. This was taken as general confirmation of the importance of these points in defining the shapes, as originally specified by Attneave [6,7].

We have been able to derive measures of shape attributes that correspond to most of the Attneave [7] indices. Attneave’s complexity index – P^2^/A – is defined as the square of the number of dots in the perimeter of the shape divided by the total dots contained within the resulting shape (including the perimeter). We derived a measure of curvature as the mean of local curvature scores for incoming and outgoing tangents across successive 5-dot subsamples. We also counted the number of local maximum curvature scores to reflect the number of major inflections that were present in each shape. Finally, symmetry was quantified by comparing mean dot distance on each side of an axis crossing the centroid, across rows or columns, depending on whether the shape displayed vertical or horizontal symmetry.

These four measures of shape attribute -- complexity, mean curvature, inflection count, and symmetry -- were assessed in 338 shapes, these being the ones that did not have internal contours substantial enough to compromise the calculation of the respective attribute indices. These measures for each of the shapes are given in Additional file 1: Table SA.

**Figure 10:**
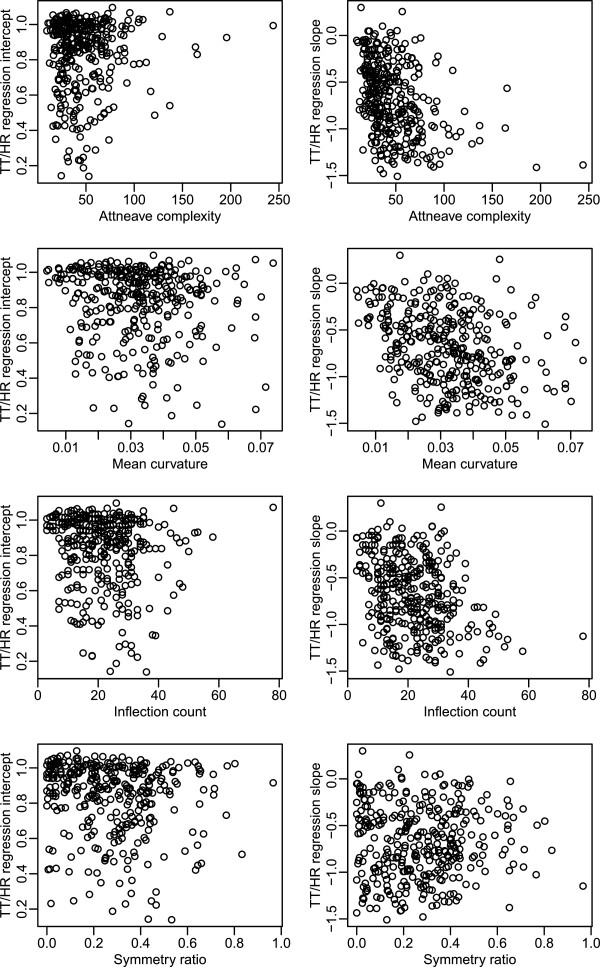
**Panels on the left show the values for the intercepts for 338 shapes, plotted against (from top to bottom) Attneave’s complexity index, mean curvature, number of inflection markers, and the index of symmetry.** Panels on the right show the size of the slope for these shapes against the same shape attributes. The complexity index does not have physical units. Curvature is specified in radians, counts are without units, and the Symmetry Ratio is specified as normalized spans from the central axes of the shapes.

We examined the degree that each of these shape attributes determines the TT regression lines for recognition. Figure 10 plots the value of each attribute for each shape against intercept (first column) and against slope (second column). None of these plots displayed a clear pattern of influence, suggesting that none of these attributes have a strong relationship with slope or intercept.

The data represented in Figure 10 were evaluated using regression techniques, with results summarized in Table 2. For intercept, none of the shape attributes “explained” more than 2.1% of the total variance individually, and a regression with all four attributes as predictors gave a multiple R^2^ value of 4.1%. Considered individually, all attributes except symmetry “explained” roughly 10% of the variability in slope; taken together in one regression model, the total variability explained is 20.0%. The usual 5% standard of statistical significance was met for all relationships except two: between intercept and complexity; and between slope and symmetry. As evident in the plots and supported by the relatively low R^2^ values, these relationships are rather weak, so we conclude that their significance is primarily a consequence of the large number of shapes. Evaluation of this many shapes allows a fairly weak relationship to register as significant.

**Table 2:** Although there is a significant correlation between most of the shape attributes and the two measures of the regression line, the percent variability “explained” by these relationships is generally fairly low, with all four attributes together accounting for only 4.1% and 20.2% of the variability in intercepts and slopes respectively

	**Intercept**		**Slope**	
**Attribute**	**R**	**R**^**2**^	**R**	**R**^**2**^
complexity	0.041	0.2%	-0.368	13.6
mean curvature	-0.132	1.7%	-0.334	11.1%
inflection count	-0.123	1.5%	-0.310	9.6%
symmetry	-0.143	2.1%	0.007	0.0%
Multiple R^2^		4.1%		20.0%

## Discussion

**Figure 11:**
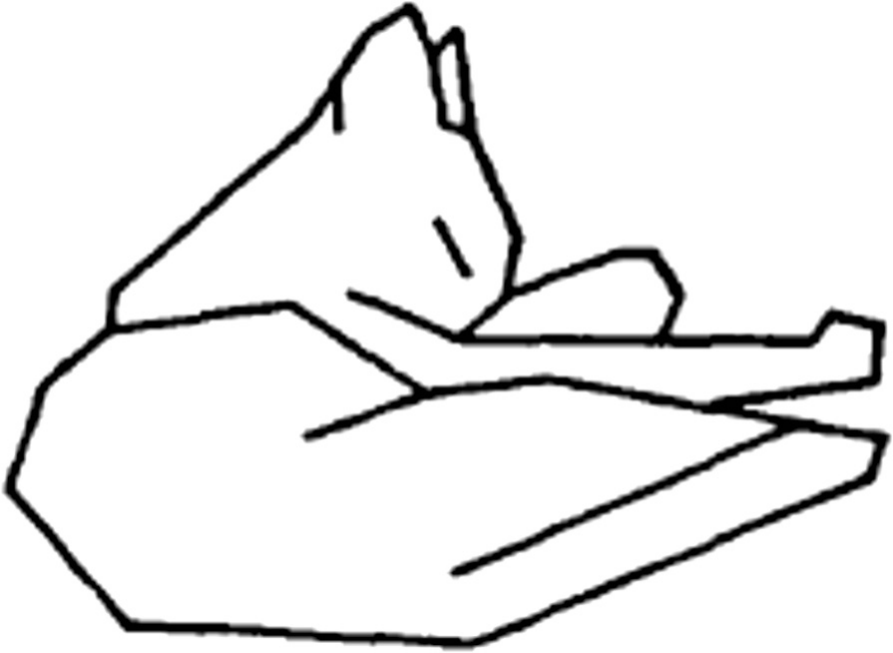
**Attneave **[6]** asserted that sharp curves and corners provided information that was most critical for shape recognition.** He illustrated this concept with a drawing in which those points were connected by straight-line segments.

As outlined above, Attneave [6] proposed that corners and sharp curves provide critical shape information, and that we identify shapes on the basis of this information. To illustrate this point, he marked the major inflection points on an outline drawing of his cat and then used straight segments to connect these points, as illustrated in Figure 11. The fact that one can recognize this representation as a sleeping cat was offered, and is generally accepted, as evidence in support of his proposition.

**Figure 12:**
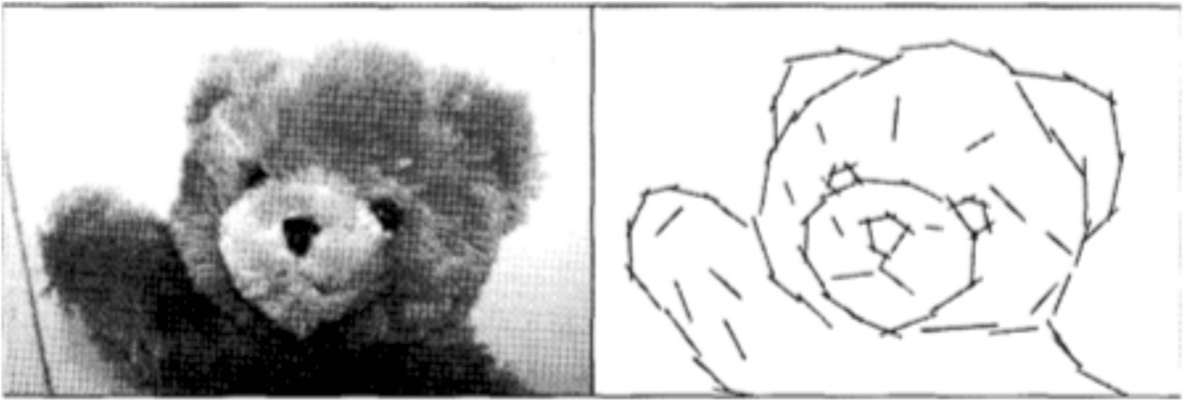
Computational theories for shape recognition often begin with specification of attributes such as the orientation and length of contours (after Marr & Nishihara, ref. 16).

The problem with this demonstration is that it does not unambiguously support the point that Attneave was advancing. One could just as well conclude that the location, orientation, and length of the straight segments themselves provide the essential cues for identifying the shape. This would certainly be consistent with an extensive body of neurophysiological evidence that cortical neurons function as selective filters for oriented lines and edges [18,19], and see [24] for a review of related findings. It also aligns with machine vision concepts, such as those advanced by Marr & Nishihara [25] who argued that “edge assertions” provide essential shape cues (see Figure 12).

In fact neither Attneave, nor many that followed his lead, actually tested whether the straight segments or the curved ones contributed most to recognition of shapes. He did provide evidence that more highly curved and angular shapes were judged to be more complex [7]. It was simply assumed that it would be more difficult to identify a complex shape. The results reported above show that complex and simple shapes alike can be identified readily as long as the contour markers are presented simultaneously.

Attneave [6,7] also demonstrated that when subjects were asked to place dots in an effort to reproduce amorphous or random-generated shapes, the dots are more frequently placed at the major points of inflection. This was replicated by Norman et al. [21], and De Winter & Wagemans [23] found the same result when subjects were asked to use the dots to mark significant boundary points on drawings of everyday objects. The decision of where to place the dots could indicate judgments of how elemental parts could be assembled to construct the object or how one would draw it. Alternatively or in addition, these decisions may just reflect intuitions that correspond to the widely held belief that recognition is based on segment attributes.

The present evidence provides very little support for the proposition that the shape attributes that we quantified contribute to efficacy of recognition. The most telling result was that measures of complexity and curvature were at best very weak predictors for intercept values for the inventory of shapes. For the relation of Attneave’s complexity measure to intercept, the R^2^ value was 0.2%, for mean curvature it was 1.7%, and for inflection count it was 1.5%.

Though it was most highly correlated with intercept values, the role of symmetry itself was minimal, and what relationship was present could well relate more to familiarity with symmetrical exemplars than to the shape encoding process, *per se*. A great many objects are represented in drawings and photographs at an orientation that emphasizes symmetry. These are often described as “canonical” views of the object, this being the perspective that provides the least ambiguity with respect to depth or that is least likely to hide parts that help define the object. We expect that the symmetrical object will be easier to identify, and what is surprising here is how weak this factor is as a predictor of hit rate when all the boundary markers are displayed simultaneously for only 50 microseconds. We believe that this may call for a complete re-examination of assumptions about the nature of shape encoding.

The finding that complexity and curvature indices have some relationship to slope may be explained in terms of redundancy of neighboring dots. As discussed above, the brief and successive display of dots allows for recognition to the extent that the information can be integrated before it decays. Recognition of a relatively simple shape is possible when only a small portion of the dots have been displayed, but a more complex shape requires a much larger portion. The requirement to integrate information over time creates the condition where the amount of redundancy is most critical to recognition, and this is reflected in the slope that is observed. No matter how complex the shape, the nervous system can register and encode the information needed for recognition as long as a simultaneous critical density of dots is available. However, when the dots are displayed successively, the density required for recognition become more important, and thus complex shapes will manifest a steeper slope.

## Conclusion

Many common objects can be identified when they are being represented by an array of dots that lie along their outside boundary. High levels of recognition are possible even when those dots are shown for a very brief period of time, when only a sparse sampling of the dots is displayed, and when the dots are shown successively, each for a very brief interval.

The present experiment determined the hit rate for each of 376 shapes when all the dots were shown simultaneously for 50 microseconds. There was no indication that difficulty of recognition of a given shape was a function of how many dots were needed to represent the border.

When the dots were shown successively, recognition declined as a function of the total time that was required to display the dots, and the decline appeared to be approximately linear for each of the shapes. There was evidence that the observed slope for a given shape is determined by the amount of redundancy that is present among neighboring dots.

A number of theorists have suggested that physical attributes of the boundary provide the basis for shape recognition. We examined a number of these attributes -- complexity of the shape, amount of curvature, number of inflection points, and amount of symmetry – to determine whether they predicted how readily the shape could be identified under the simultaneous or successive display conditions of the present experiment. None of the attributes made more than a modest contribution to shape recognition.

## Abbreviations

AlGaInP: Aluminum, gallium, indium, phosphorous; Cd: Candela; LED: Light emitting diode; Lux: Lumen per meter squared; M: Meters; Mhz: Megahertz; MIPs: Million instructions *per se*cond; μs: Microseconds; Mm: Millimeters; Ms: Milliseconds; MTDC: Minimum transient discrete cue; Ns: Nanoseconds; P: Probability; P^2^/A: Square of perimeter divided by area; R: Correlation coefficient; R^2^: Square of correlation coefficient; SOA: Stimulus onset asynchrony; T1: Display time for a given dot; TT: Total time to display all dots.

## Competing interests

The authors declare that they have no competing interests.

## Authors’ contributions

Experiments were designed by EG, and the experiments were run in his laboratory. Data analyses and plots were done by TO. Both authors contributed to the interpretation of results and writing of the article. All authors read and approved the final manuscript.

## Supplementary Material

Additional file 1 **Table SA.** The first four columns of the table provide the name for each of the 376 shapes, the number of dots marking the contours, the intercept of the regression line, and the slope of the regression line. The last four columns provide measures of contour attributes for the 338 shapes that were represented only by the outer boundary. The values reflect complexity, net curvature, number of inflections, and amount of symmetry for each of these shapes. Click here for file
