# Supplementary material for: Evaluating the contribution of shape attributes to recognition using the minimal transient discrete cue protocol
Source: Behav Brain Funct. 2012 Nov 12;8:53. doi: 10.1186/1744-9081-8-53 (PMC3537607; doi:10.1186/1744-9081-8-53)
Supplement: Additional file 1 — Table SA. The first four columns of the table provide the name for each of the 376 shapes, the number of dots marking the contours, the intercept of the regression line, and the slope of the regression line. The last four columns provide measures of contour attributes for the 338 shapes that were represented only by the outer boundary. The values reflect complexity, net curvature, number of inflections, and amount of symmetry for each of these shapes. [file 1744-9081-8-53-S1.pdf]

name	# dots	slope	int	comp	curvature	inflect #	symm
AK 47	160	-1.20	0.89	54.4	3.31	14	2.09
Africa	183	-0.07	0.87	17.6	5.16	30	6.94
Batman insignia	201	-0.74	1.07	29.6	4.18	23	0.92
California	170	-0.76	1.03	19.0	3.42	20	10.12
Eiffel tower	162	-0.50	0.41	44.3	2.62	13	1.51
Mickey Mouse	312	-1.24	0.93	55.0	5.19	52	4.58
Mickey Mouse head	201	-0.62	0.68	21.7	3.13	21	5.54
Saturn	290	-0.79	1.03	—	—	—	—
Texas	201	-0.07	1.01	19.6	2.35	17	5.90
USA	198	0.05	0.97	22.7	4.71	28	4.65
VW Beetle	190	-0.35	1.04	—	—	—	—
airplane	235	-1.03	0.92	77.1	4.07	24	4.61
alarm clock	275	-0.45	1.04	29.2	4.19	30	1.51
alligator	227	-0.60	0.53	43.7	4.43	30	9.43
alligator head	265	-1.45	0.96	—	—	—	—
anchor	209	-0.57	0.91	65.8	1.95	12	5.34
angel	239	-0.59	0.66	38.4	2.83	20	5.42
antique bathtub	260	-0.71	0.96	—	—	—	—
antique car	179	-0.28	0.62	20.5	4.01	26	4.30
antique chair	201	-0.84	0.81	30.4	2.34	16	6.79
arm chair	285	-0.97	0.99	—	—	—	—
baboon a	276	-0.59	0.87	52.5	3.08	25	6.52
baboon b	315	-0.66	0.94	71.0	3.45	30	7.18
baby bottle	146	-0.84	0.86	17.4	1.80	12	0.18
backhoe	291	-0.77	0.50	68.8	3.72	37	6.00
badge	159	-0.80	0.78	12.3	1.94	11	0.51
ballerina	231	-1.08	1.10	77.4	3.70	26	2.04
banana	179	-0.45	1.02	23.1	0.78	5	12.84
banana bunch	389	-0.41	1.03	73.8	1.39	16	2.97
baseball catcher	252	-0.90	0.85	33.4	4.43	34	8.40
baseball pitcher a	260	-0.76	0.97	65.8	2.73	25	5.59
baseball pitcher b	242	-0.79	0.70	58.4	3.84	31	2.81
baseball player	224	-0.73	1.00	55.0	3.25	24	3.89
basketball player	255	-0.75	0.69	65.0	3.49	27	4.81
bat	155	-0.95	1.02	30.6	6.24	27	4.30
bear	212	-0.77	0.81	29.4	3.17	22	5.60
bee	308	-0.32	0.47	65.3	3.61	30	5.53
beetle	268	-0.84	1.01	53.4	5.00	32	2.46
bell	155	-0.47	0.99	29.0	1.80	10	0.61
binoculars a	175	-0.87	0.85	19.2	2.66	18	3.10
binoculars b	408	-0.82	1.00	—	—	—	—
bird talon	294	-0.44	0.30	50.9	3.37	29	7.45
blender	207	-0.71	0.55	28.5	3.06	21	3.30
bongo drums	295	-0.72	0.55	—	—	—	—
boot	182	-0.37	1.03	19.4	2.36	15	6.80
bottle	142	-0.38	1.02	23.3	0.79	4	0.00

name	# dots	slope	int	comp	curvature	inflect #	symm
bow tie	137	-0.43	0.43	19.1	1.82	11	0.63
bowler	273	-0.70	0.79	59.8	3.64	31	6.43
bowling pin	133	-0.43	0.94	19.9	0.47	3	0.14
boxer	342	-1.22	0.99	—	—	—	—
briefcase	261	-0.80	1.01	—	—	—	—
buffalo	237	-0.78	0.98	33.3	4.13	29	6.05
bull	301	-1.13	0.96	71.3	4.47	40	7.46
bull fighter a	192	-0.81	0.59	22.8	3.96	23	4.90
bull fighter b	249	-0.59	0.36	44.0	3.68	28	5.80
burro	358	-0.98	0.80	89.9	2.68	29	10.42
butterfly a	180	-0.33	1.00	20.2	3.91	22	9.00
butterfly b	305	0.00	1.00	45.3	3.44	32	3.07
camel	281	-0.42	1.01	40.4	2.98	28	6.05
campfire	421	-0.48	0.28	—	—	—	—
candelabra	311	-1.16	0.93	129.0	6.49	53	3.41
candle	216	-0.83	0.80	55.7	3.77	22	3.97
candy dish	235	-0.16	0.23	42.4	1.85	18	0.28
cap	157	-0.20	0.94	16.1	0.79	6	5.92
car	135	-0.15	1.00	21.9	2.57	14	1.78
carrot	165	-0.77	0.51	33.4	4.66	22	13.32
cat	247	-1.02	0.65	39.8	3.24	23	4.92
caterpillar	171	-0.36	0.22	31.7	6.84	33	7.46
ceiling fan	268	-0.70	0.67	92.4	2.92	23	2.78
centaur	396	-1.03	0.62	117.0	4.12	48	4.59
cheerleader a	225	-1.28	0.98	77.8	4.66	31	1.64
cheerleader b	269	-1.07	1.03	105.0	4.17	31	1.11
chick	176	-0.69	0.96	24.7	3.43	19	6.55
cordless drill	239	-0.48	0.70	31.1	1.90	20	7.30
Christmas light bulb	189	-0.54	0.33	—	—	—	—
Christmas tree	189	-0.83	1.05	25.1	7.38	35	2.01
church	224	-1.29	0.79	35.1	3.13	21	2.57
claw hammer	167	-0.90	0.97	55.1	2.07	10	3.09
clock	257	-0.79	0.64	—	—	—	—
coat	270	-0.51	0.98	30.8	2.02	19	0.96
coat hanger	159	-0.58	0.99	39.0	2.40	11	0.24
cockatoo	228	-0.23	0.14	53.0	5.80	36	8.52
cockatoo head	302	-0.50	0.29	59.9	4.11	31	5.76
coffee pot	241	-0.79	0.85	23.8	1.30	12	6.79
coke bottle	152	-0.25	0.94	20.6	1.37	8	0.00
cow	255	-0.86	1.01	43.4	2.26	16	6.56
cowboy	227	-0.99	0.88	27.8	2.65	18	3.46
cowboy boot	188	-0.04	0.99	19.0	0.85	7	6.67
cowboy hat	274	-0.71	0.99	—	—	—	—
crab	357	-1.38	1.07	95.5	4.78	45	3.27
crane bird	217	-0.24	0.87	66.4	2.54	17	6.42
crescent wrench	147	-1.12	0.89	43.6	2.21	10	0.59

name	# dots	slope	int	comp	curvature	inflect #	symm
crown	339	-0.54	1.06	—	—	—	—
crucifix	192	-0.34	1.02	53.2	3.90	26	0.29
Cupid	311	-1.27	0.90	69.3	4.81	46	5.45
dagger	182	-0.50	1.01	44.3	3.18	20	12.31
decanter flask	147	-0.50	0.54	23.8	1.39	7	0.15
deer	352	-0.29	0.99	91.5	3.72	34	10.40
deer head	399	-0.81	0.49	121.2	4.23	43	3.98
desk lamp	222	-0.70	0.79	83.8	1.62	12	5.49
diamond	126	-0.05	0.96	9.5	1.21	5	0.00
dinosaur a	262	-0.23	0.56	62.4	3.43	26	10.28
dinosaur b	208	-0.50	1.01	52.3	2.52	17	10.32
dinosaur c	215	-0.71	1.05	50.4	4.55	27	6.41
dog a	279	0.05	0.89	51.3	3.21	28	5.68
dog b	234	-0.70	0.87	41.5	3.64	28	8.37
dolphin	157	-0.62	0.98	33.6	2.69	10	4.18
dragon	300	-1.10	0.64	73.2	5.03	47	3.23
dragonfly	245	-0.95	0.99	56.2	2.49	14	6.10
dress	194	-1.16	0.94	24.8	2.22	15	0.46
dress form	166	-1.01	0.97	52.4	5.14	21	0.34
duck a	171	0.02	0.85	28.5	1.90	10	3.05
duck b	251	-0.41	0.63	38.2	3.63	24	10.70
duck head	199	-0.06	0.69	21.4	1.49	10	5.99
dumbbell	184	-0.32	0.96	18.4	1.22	9	11.36
dump truck	207	-0.87	0.63	39.6	3.56	25	4.97
eggplant	193	-0.85	0.97	—	—	—	—
elephant	260	-0.84	0.97	38.4	2.86	23	6.28
emu	239	-0.28	0.93	56.7	2.66	20	7.00
eye	726	-1.30	1.03	—	—	—	—
faucet	227	-0.69	0.45	37.7	3.70	30	6.89
feather	233	-0.41	0.25	—	—	—	—
femur bone	149	-1.27	0.91	41.0	3.06	15	1.37
fencing	212	-0.94	0.76	76.2	3.36	22	6.25
fighter jet	239	-0.07	0.86	49.5	2.78	18	7.21
fire extinguisher	292	-0.65	1.00	42.7	1.69	17	6.38
fire hydrant	183	-0.96	0.90	29.2	4.83	27	1.17
fish (angel)	250	-0.23	0.60	41.8	2.78	19	5.13
fish (bass)	155	-0.39	0.78	29.2	3.86	18	2.59
fish (tropical)	197	-1.14	0.88	28.5	3.49	19	2.07
flag	297	-1.06	0.89	45.1	2.88	29	6.57
flask	230	-0.59	0.42	15.9	1.30	12	0.09
fly	351	-1.00	1.01	104.3	3.80	31	1.27
flying duck	239	0.03	0.23	44.2	2.48	18	5.36
flying pheasant	228	-0.33	0.59	42.1	4.81	28	6.76
foot and ankle	221	0.00	0.99	27.3	0.94	8	7.64
football quarterback	245	-0.40	0.87	29.8	3.16	24	8.14
formula race car	148	-0.86	0.96	32.0	4.58	21	1.87

name	# dots	slope	int	comp	curvature	inflect #	symm
four leaf clover	258	-0.59	1.01	32.9	4.36	35	6.52
fox	244	-0.97	0.89	62.0	4.31	29	3.64
freight truck	187	-1.28	0.98	34.3	4.08	23	1.63
frog	370	-0.89	0.88	65.9	5.06	49	5.62
giraffe	352	-0.37	1.03	109	2.69	27	9.62
giraffe head	185	-1.33	0.94	41.9	3.17	16	3.13
glasses	214	-0.80	1.02	70.6	2.34	16	3.20
glove a	201	-1.34	1.02	28.7	2.96	16	2.65
glove b	215	-1.05	1.06	39.7	2.38	13	3.82
goat	270	-0.82	0.57	40.3	5.63	45	6.21
golfer teeing off	257	-0.34	0.52	73.8	3.68	27	4.31
goose	163	-0.62	0.84	29.0	4.12	22	4.88
gorilla	250	-0.38	0.72	25.8	2.13	20	6.30
gramophone	229	-1.06	0.90	31.1	2.78	22	6.99
grenade	242	-0.21	0.25	30.7	3.43	31	7.13
guitar	150	-0.52	1.05	29.6	1.77	11	0.73
gun	170	-0.60	0.94	29.9	2.95	17	7.84
hammer (ball peen)	159	-1.29	1.05	60.2	2.58	12	0.48
hammerhead shark	242	-1.38	0.97	48.1	3.90	27	10.42
hand and finger	262	-0.46	1.01	48.4	2.70	24	3.42
hand shovel	143	-0.87	0.98	37.9	2.00	8	0.13
hand with peace sign	207	-0.85	1.05	32.2	2.20	14	2.69
handcuffs	272	-1.03	0.73	63.2	3.11	31	12.25
hat	160	-0.15	1.02	17.1	1.20	6	2.59
hatchet	165	-0.93	0.72	58.5	2.38	13	3.07
hawk	337	-0.94	0.65	67.0	4.49	33	6.94
headphones	273	-0.30	1.03	70.6	1.35	15	0.59
heart	170	-0.07	1.01	10.8	0.42	3	2.33
hedge shears	227	-0.66	0.53	78.7	1.64	9	2.33
helicopter	229	-1.39	0.94	86.3	2.93	20	1.36
helmet	160	-0.38	0.97	13.4	0.75	4	3.08
hen a	191	-0.32	0.92	27.8	4.52	27	6.92
hen b	217	-0.19	1.00	39.6	4.24	25	4.71
hippo a	254	-0.26	0.28	32.5	3.33	27	2.91
hippo b	208	-0.57	0.47	28.1	3.77	26	4.57
hippo head	222	-0.16	0.14	24.4	2.93	24	6.92
hockey goalie	314	-1.05	0.68	49.7	3.83	40	5.12
hockey player	207	-1.25	0.86	47.7	4.54	27	5.86
horse a	289	-1.01	0.73	74.6	3.40	27	5.34
horse b	347	-0.03	0.98	75.8	3.16	31	10.49
horse head	196	-0.74	0.78	22.3	3.40	19	6.70
horseshoe	272	-0.15	1.02	56.5	0.47	5	0.84
hot air balloon	168	0.30	0.53	14.0	1.75	11	0.40
hourglass	242	-0.10	0.89	37.8	2.18	16	0.20
house	206	-1.23	1.01	16.0	2.66	14	3.35
human skull	174	-0.71	1.01	12.6	1.87	13	4.27

name	# dots	slope	int	comp	curvature	inflect #	symm
hummingbird	161	-0.21	0.80	34.7	2.08	9	7.15
hunting knife	145	-1.21	0.86	37.2	4.23	13	0.62
ice cream cone	148	-0.36	0.77	16.2	2.38	13	0.97
ice skate	297	-0.87	0.99	43.7	1.99	22	5.90
ice skater	307	-0.35	0.35	73.8	4.44	39	4.96
indian with headdress	235	-0.63	0.35	18.6	7.15	38	3.69
infant crawling	237	-1.04	0.75	42.6	4.14	30	4.74
infant walking	236	-0.82	0.87	42.4	5.16	35	1.33
iron	239	-0.57	0.72	25.7	1.97	17	4.36
jackrabbit	244	-0.30	1.01	35.3	2.83	21	4.98
Japanese temple	240	-1.17	0.76	39.2	4.13	30	0.69
jaybird	198	-0.35	0.50	37.6	5.22	28	7.99
jellyfish	488	-0.57	0.83	165.3	2.62	35	3.32
kangaroo a	212	-0.99	0.92	62.1	3.30	19	8.76
kangaroo b	245	-0.91	0.88	69.8	4.08	26	10.7
karate kick	242	-1.16	0.94	46.8	3.75	28	4.55
kerosene lamp	157	-0.14	0.43	28.0	3.68	18	0.20
key	145	-0.88	0.86	25.0	4.29	23	0.41
kite	198	-1.27	0.86	65.6	7.01	27	6.95
knife	132	-1.20	0.94	49.1	1.52	5	0.71
large bow	249	-0.49	1.03	37.9	3.32	19	0.82
large hook	243	-0.75	0.84	41.3	1.67	15	2.06
leaf	258	-0.15	1.00	51.4	5.92	33	3.38
Liberty Bell	313	-0.50	0.84	43.8	3.68	39	0.60
light bulb	205	-0.96	0.98	—	—	—	—
lion	282	-1.31	0.99	59.6	4.34	37	5.98
lips	248	-0.16	1.00	—	—	—	—
lizard	241	-0.84	1.01	59.5	2.88	19	7.91
long horn cattle skull	218	-0.09	0.92	59.6	3.38	22	1.46
macaw	157	-0.98	0.86	34.0	3.28	15	5.54
magnifying glass	223	-0.66	0.84	—	—	—	—
mail box	281	-0.64	1.03	—	—	—	—
man	248	-0.45	1.04	69.3	3.40	22	0.69
mariachi	326	-1.19	1.00	67.2	3.23	29	5.15
meat cleaver	151	-1.03	0.87	31.3	1.57	8	1.93
mens lavatory symbol	334	-1.07	0.61	—	—	—	—
mermaid	277	-0.79	0.49	68.8	4.17	33	4.02
microscope	287	-1.32	0.82	82.1	3.88	32	6.29
mission	396	-0.63	0.86	55.7	3.37	36	1.20
monkey	255	-0.82	0.92	72.9	4.25	30	4.79
moth	256	-0.05	0.98	39.9	3.21	25	2.22
motor scooter	227	-0.43	0.88	42.4	3.69	23	4.09
motorcycle (chopper)	225	0.26	0.82	56.7	4.81	31	3.60
motorcycle a	197	-0.67	1.01	31.2	5.36	30	4.88
motorcycle b	238	-0.13	0.95	41.8	3.86	26	3.38
mug	298	-0.46	1.01	26.4	1.24	14	3.62

name	# dots	slope	int	comp	curvature	inflect #	symm
mushroom	186	-0.39	0.88	23.0	1.39	10	1.33
music stand	192	-1.04	0.99	62.3	2.69	13	2.44
office chair	263	-0.74	0.98	66.5	4.83	35	3.29
oil can	223	-0.54	0.45	58.9	2.21	16	10.31
open ended wrench	158	-1.33	0.94	62.1	3.13	15	0.38
ostrich	243	-0.05	0.88	70.0	3.01	23	7.73
owl	210	-0.98	0.76	30.5	4.76	26	3.67
Pac Man	197	-0.07	0.97	15.5	0.68	3	1.58
padlock	262	-0.52	0.40	40.7	2.33	20	3.42
paint brush	175	-0.89	0.99	—	—	—	—
paint roller	182	-0.40	0.42	51.6	2.79	15	10.15
pan	150	-0.39	0.63	18.2	1.95	13	5.11
passenger plane	242	-0.79	1.04	56.4	3.97	27	5.61
pear	145	-0.36	0.96	17.9	0.82	4	1.61
pegasus	313	-1.13	0.82	71.5	6.27	50	4.35
pelican	252	-0.95	0.92	43.3	3.49	24	7.78
penguin	221	-0.64	1.04	32.2	3.31	18	2.10
pepper	155	-0.64	0.69	22.5	1.64	8	6.44
perfume bottle	203	-0.52	0.60	20.3	1.41	10	6.65
phone a	153	-0.62	0.98	26.8	2.07	11	8.62
phone b	279	-0.14	0.98	—	—	—	—
piano a	297	-0.88	1.02	47.8	3.92	35	6.46
piano b	235	-1.23	0.79	27.5	3.5 0	23	6.38
picket fence	244	-1.39	0.99	244.0	3.50	29	0.91
pickup (vintage)	178	-0.42	0.66	26.7	4.99	27	2.44
pickup truck	213	-0.59	1.03	—	—	—	—
pig	219	-0.77	1.00	35.3	3.70	20	5.32
piggy bank	214	-0.41	0.43	20.8	3.37	21	5.50
pineapple	220	-0.47	0.63	38.4	6.81	29	1.16
pipe	150	-0.45	1.04	44.7	1.31	6	8.46
pitch fork	252	-0.97	0.54	136.9	2.57	17	3.49
pitcher	244	-0.71	1.00	20.1	1.47	12	6.59
pliers a	224	-1.28	0.98	97.1	1.65	9	0.42
pliers b	237	-0.95	0.77	99.4	1.74	11	1.26
polar bear	188	-0.57	0.72	23.9	2.35	15	7.09
porpoise	167	-0.46	0.96	32.4	3.91	17	5.87
pot	176	-0.14	0.94	17.7	3.27	16	1.15
potted plant	241	-1.14	0.84	71.5	5.58	37	3.40
power boat	198	-0.72	0.82	31.1	3.39	21	4.98
quail	222	-1.04	0.70	43.8	5.17	29	6.01
rabbit	215	-0.09	0.97	29.9	2.70	16	7.60
ram	391	-0.82	0.97	90.9	3.52	42	8.60
rat	191	-0.76	0.88	46.5	4.23	20	11.44
rhino a	186	-0.83	0.99	27.7	3.62	21	4.10
rhino b	225	-1.09	0.92	34.9	3.85	22	4.73
rhino head	239	-1.09	0.72	26.1	3.09	23	5.81

name	# dots	slope	int	comp	curvature	inflect #	symm
rifle a	166	-0.97	0.99	87.5	3.01	11	1.90
rifle b	179	-1.15	1.02	67.9	3.48	15	8.14
roadrunner bird	230	-0.85	0.68	74.5	6.1	30	7.05
rocking horse	319	-0.60	1.03	65.5	2.91	26	4.16
rollerblade	396	-0.65	1.04	—	—	—	—
rolling pin	138	-1.43	0.87	34.0	2.43	10	0.00
rooster a	248	-0.17	0.91	42.3	4.11	25	8.89
rooster b	272	-0.83	0.90	52.8	5.02	33	8.02
rose	221	-0.81	0.64	57.1	5.2	31	9.95
runner	259	-1.19	0.86	52.5	3.98	34	4.97
russian folk dancer	228	-0.27	0.19	47.4	4.25	26	4.48
sailboat a	439	-0.22	1.02	93.7	1.45	16	0.51
sailboat b	209	-0.94	0.90	43.3	2.29	13	2.49
salamander	201	-1.41	0.94	89.2	5.64	29	3.04
saxophone	235	-0.82	0.71	57.1	1.47	10	7.00
scimitar	162	-1.17	0.97	59.9	3.37	13	4.38
scissors	303	-1.15	1.02	77.4	3.15	31	3.04
scorpion	360	-1.41	0.93	195.8	5.2	44	2.98
scuba diver	262	-0.62	0.36	—	—	—	—
sea horse	171	-1.00	0.91	46.7	5.24	27	1.47
sea lion	201	-0.63	0.52	24.1	2.1	13	5.94
sea turtle	225	-0.75	0.79	25.0	2.91	21	7.03
senorita dancing	254	-1.10	0.88	60.9	5.03	34	4.19
shark	184	-0.41	0.94	40.7	3.24	14	1.83
ship propeller	261	-0.33	0.88	40.9	1.37	11	5.60
shoe (man's)	156	-0.20	0.98	20.3	2.23	12	2.20
shoe (woman's)	163	-0.37	1.03	30.3	1.15	6	6.66
shorts	191	-0.79	1.05	15.8	1.47	7	2.12
sickle	175	-0.91	0.68	64.7	1.14	7	5.80
single handle faucet	255	-1.23	0.80	34.3	2.38	17	6.62
skateboarder	277	-1.23	1.05	—	—	—	—
skier (down hill)	252	-1.30	0.86	85.5	3.22	23	6.80
slipper	138	-0.48	0.50	22.9	1.29	7	1.66
snail	175	-0.57	0.62	31.0	2.84	13	3.04
snake	172	-1.36	1.06	72.7	2.93	18	5.15
snowflake	431	-1.13	1.07	136.9	6.84	78	1.40
snowman	252	-1.31	1.00	—	—	—	—
soccer player	305	-0.67	0.46	54.7	3.21	33	10.55
sock	143	-0.21	0.90	24.8	0.99	7	3.98
sombrero	162	-0.23	0.96	23.2	0.84	4	0.95
space shuttle	320	-0.60	0.39	—	—	—	—
spade	239	-0.30	1.03	31.5	2.02	13	0.72
spider	476	-0.99	0.87	163.5	3.79	47	3.18
spoon	133	-0.91	1.04	42.5	1.67	11	0.08
spray bottle	179	-0.90	1.01	31.0	2.59	14	2.10
spur	159	-0.44	0.25	47.2	5.03	15	1.82

name	# dots	slope	int	comp	curvature	inflect #	symm
squirrel	244	-0.48	0.90	30.2	2.67	23	3.56
stapler	212	-0.92	0.61	49.7	1.41	11	1.52
star of David	199	0.05	0.97	21.3	2.95	13	0.12
starfish	210	-0.15	1.02	33.9	1.69	15	5.16
steam train	337	-1.29	0.90	48.9	5.37	58	4.48
strawberry	240	-1.41	1.05	—	—	—	—
submarine	146	-0.56	0.69	27.7	3.10	15	2.90
surfer	317	-1.32	0.96	—	—	—	—
surfing	277	-1.23	0.84	90.1	3.38	26	2.88
swan	254	-1.48	1.06	37.1	2.24	17	4.52
swordfish	199	-0.24	0.83	66.8	3.27	17	3.17
syringe	160	-1.15	0.92	29.9	4.75	23	15.45
table a	276	-0.37	0.88	50.8	3.24	29	3.71
table b	288	-0.32	0.91	61.1	2.00	19	3.32
table c	251	-0.14	0.64	56.1	2.97	21	4.65
table lamp a	184	-0.44	1.05	22.8	2.02	10	0.29
table lamp b	183	-0.22	1.02	28.2	2.22	12	0.54
tank	185	0.10	0.41	36.3	2.60	14	2.84
tea pot	206	-0.53	1.03	28.6	3.16	20	3.36
teacup	224	-0.87	0.94	31.4	3.08	21	4.06
teddy bear	237	-0.99	0.97	35.8	3.73	30	3.27
telescope	238	-0.78	0.87	89.5	2.60	18	6.38
tennis player	273	-1.00	0.84	74.6	4.59	41	5.72
tennis player serving	244	-1.35	0.88	46.1	3.18	26	5.46
tiger	235	-0.50	0.81	53.6	4.44	31	3.45
toad	245	-0.83	0.83	32.7	4.37	26	2.79
toilet	224	-0.73	0.76	21.8	1.65	11	8.70
tooth	217	-0.09	0.96	30.5	1.02	9	0.43
toothbrush	198	-1.24	0.78	105.4	4.36	16	2.26
top hat	178	-0.05	0.83	16.3	1.22	8	0.06
tractor a	229	-0.78	0.58	26.4	3.80	28	4.60
tractor b	237	-0.83	0.68	30.1	5.12	32	5.40
trident	180	-1.08	0.79	112.5	2.58	12	0.00
trumpet	215	-1.51	0.98	51.6	6.22	34	2.05
tulip	229	-1.14	1.04	70.0	3.95	25	4.71
turkey	255	-1.08	0.75	25.1	6.84	43	5.69
turtle a	170	-0.56	0.95	26.3	4.18	19	3.34
turtle b	201	-0.13	0.89	—	—	—	—
umbrella	198	-0.35	0.95	22.2	2.55	14	7.11
vase	163	-0.15	1.02	17.0	1.51	8	0.21
Viking hat	271	-0.45	0.47	37.0	1.63	15	3.96
violin	173	-0.56	0.84	37.4	6.29	29	0.43
walking pheasant	223	-0.94	0.85	66.1	3.80	24	11.39
watering can	193	-0.61	0.61	26.0	1.83	15	2.78
whale	184	-0.35	0.38	—	—	—	—
wheelbarrow	212	-0.55	0.51	40.0	2.77	16	5.20

name	# dots	slope	int	comp	curvature	inflect #	symm
windmill	242	-1.20	0.97	44.0	3.77	24	2.16
wine glass a	233	-0.15	1.02	26.0	1.11	9	0.46
wine glass b	175	-0.07	1.01	30.7	1.58	10	0.24
wolf	266	-0.50	0.96	49.1	2.98	22	6.09
women's lavatory symbol	336	-1.35	0.98	—	—	—	—
yin yang symbol	333	-0.12	0.96	—	—	—	—
